# Annotations

**Published:** 1908-06-20

**Authors:** 


### Annotations.

### The Proposed Bombay Medical Congress.

In December 1904 a very successful Indian Medical Congress was held at Calcutta. It is now proposed to hold a second Congress towards the end of February next in Bombay. A preliminary meeting has been held, under the presidency of the Governor of Bombay, and a Central Committee has been formed. The proposal has aroused much enthusiasm among the members of the medical profession resident in and around the Bombay district, and the claims of Bombay as a fitting centre for the second Indian Medical Congress seem to be generally recognised throughout the profession in India. The Central Committee, of which Lieut.-Col. W. E. Jennings, of the editorial staff of the *Indian Medical Gazette*, is general secretary, has already drafted the main outlines of the work of the Congress. In the papers, discussions, and exhibits special prominence will be given to the following subjects—namely, The Ætiology, Prophylaxis, and Treatment of Plague, Enteric Fever and Relapsing Fever; the Differential Diagnosis of the various types of Malarial Fever, with suggestions as to means of prevention and exhibition of the results of past measures from available statistics; the part played by Parasitic Insects (other than fleas and mosquitoes) in the dissemination of diseases peculiar to the Tropics, with suggestions as to the best means of obviating the attacks of those insects; the Pathological conditions dependent upon the invasion of the Leishman-Donovan body, with suggestions as to treatment and prophylaxis; the Ætiology and Differential Diagnosis of the various clinical types of Dysentery—their treatment and prophylaxis; the Treatment of Cholera; and Sanitation as applied to India.

### "Crowner's Law."

The coroner for South-West London has the courage of his convictions, however opposed to common-sense they may appear to medical men. With regard to his latest mare's nest, which we dealt with in our last issue, Mr. Troutbeck has been well snubbed in a trenchant leading article in last Friday's *Times*; but so far from being persuaded of the unwisdom of the course he has pursued, the coroner-militant returns to the attack and accuses that paper of personal bias. As a vindication of his actions in holding an inquest on the body of a patient from whom a cerebral tumour was removed three days before her death, this letter is singularly unconvincing and irrelevant. Indeed, the only statement worth noticing is that in which he disclaims any intention to cast a slur upon the medical profession, a disclaimer the more necessary in that there is little *primâ facie* ground for suspecting that sentiment from his actions. But the letter is quite remarkable for that which it omits or ignores, if not for that which it contains. The very serious specific allegations of Dr. McManus, one of the direct representatives of the profession on the General Medical Council, are quite unanswered. The reason given for this is that they were refuted in a letter to the Lord Chancellor in 1903, the very year in which that official expressed strong disapproval of Mr. Troutbeck's proceedings! The gross mistake of the pathologist selected to perform the *post-mortem* examination in this case by Mr. Troutbeck, which was exposed by Sir Victor Horsley in the most damaging manner, was made the subject of the first paragraph of the coroner's first letter to the *Times* on June 10. That paragraph imputed to Sir Victor in thinly-veiled terms a clinical blunder and an incorrect statement at the inquest. Having been thus betrayed into a totally false position by the error of his own pathologist, does Mr. Troutbeck apologise to Sir Victor? Not a bit of it; but he inserts in a postscript a grudging admission that he has "no reason to doubt that Sir Victor Horsley was perfectly able to give full information of the clinical facts to the jury." In the same postscript he admits that his informant about the case was the Registrar of Deaths; but he omits to say that this was by his own order, and he omits to explain how it happens that action of this sort is taken for the first time in connection with the surgeon who was most prominent in bringing to notice the actions which in 1903 incurred the censure of Lord Halsbury.

### The Opponents of our Profession.

Mr. Stephen Paget, the indefatigable Hon. Secretary of the Research Defence Society, deserves the thanks, not only of the medical profession, but also of everybody who has at heart the welfare of the human race and the cause of justice and truth. We have already drawn attention to the work and methods of this society, and have pledged ourselves to support its principles and help in the up-hill task which it has set itself. Our readers, it is true, are not personally in need of constant reminders of the value and the necessity of experiments upon animals. But prejudice and calumny are still hard at work and it is our duty to confront our opponents at every point, and educate the public in the true facts of medical research. The Research Defence Society grows daily in membership and influence, and its active campaign has already opened. Wherever now the anti-vivisectionists appear their specious rhetoric and cooked statistics are met by logic, facts and common sense. The only pity is that thirty years should have passed before this method was adopted. The most valuable asset of the Society is, of course, Mr. Stephen Paget, and his recent address at St. Bartholomew's Hospital, before the Abernethian Society, is likely to have far-reaching results. Speaking to a crowded and enthusiastic audience of students, medical men, and nurses, Mr. Paget discussed the various adversaries of our profession. He first exposed the false and cruel doctrines of the anti-vaccinationists and the disciples of Christian Science. But the pith of his address lay in his indictment of the anti-vivisection movement. Knowing well that an unsupported appeal to authority is wasted alike upon medical students and anti-vivisectionists, he dealt with facts alone and urged his hearers to do the same. Armed with the right weapons, the medical student is an effective adversary to the enemies of research; but only then. Mr. Paget has grasped this, and much good should therefore come of his address.

